# Exome sequencing reveals a novel *TTC19* mutation in an autosomal recessive spinocerebellar ataxia patient

**DOI:** 10.1186/1471-2377-14-5

**Published:** 2014-01-07

**Authors:** Hiroyuki Morino, Ryosuke Miyamoto, Shizuo Ohnishi, Hirofumi Maruyama, Hideshi Kawakami

**Affiliations:** 1 Department of Epidemiology, Research Institute for Radiation Biology and Medicine, Hiroshima University, 1-2-3, Kasumi, Minami-ku, Hiroshima 734-8553, Japan; 2 Department of Neurology, Tanabe Neurological Hospital, Fujiidera, Osaka, Japan

**Keywords:** Exome sequencing, Mitochondrial respiratory chain complex III, Nonsense mutation, Spinocerebellar ataxia, *TTC19*

## Abstract

**Background:**

Spinocerebellar ataxias (SCAs) are heterogeneous diseases characterized by progressive cerebellar ataxia associated with dysarthria, oculomotor abnormalities, and mental impairment. To identify the causative gene, we performed exome sequencing on a Japanese patient clinically diagnosed with recessive SCA.

**Method:**

The patient is a 37-year-old Japanese woman with consanguineous parents. The head magnetic resonance imaging (MRI) showed cerebellar atrophy and T1 low/T2 high intensity at the bilateral inferior olives. Single-nucleotide polymorphism (SNP) genotyping and next-generation sequencing were performed, and the variants obtained were filtered and prioritized.

**Results:**

After these manipulations, we identified a homozygous nonsense mutation of the *TTC19* gene (p.Q277*). *TTC19* has been reported to be a causative gene of a neurodegenerative disease in Italian and Portuguese families and to be involved in the pathogenesis of mitochondrial respiratory chain complex III (cIII) deficiency. This report is the first description of a *TTC19* mutation in an Asian population. Clinical symptoms and neuroimaging are consistent with previous reports. The head MRI already showed abnormal features four years before her blood lactate and pyruvate levels were elevated.

**Conclusions:**

We should consider the genetic analysis of *TTC19* when we observe such characteristic MRI abnormalities. Genes associated with mitochondrial function cause many types of SCAs; the mutation we identified should help to elucidate the pathology of these disorders.

## Background

Spinocerebellar ataxias (SCAs) are genetically, clinically and pathologically heterogeneous diseases characterized by progressive cerebellar ataxia variably associated with dysarthria, oculomotor abnormalities, epilepsy, and mental impairment. Neuronal loss is observed in cerebellum and brainstem pathologies, and neuroimaging demonstrates the atrophy of those regions. These pathologies can be caused by autosomal dominant, autosomal recessive, and X-linked mutations, and many of the dominant mutations are caused by CAG triplet repeat expansion. Thirteen recessive SCAs (numbered as SCAR1-13) have been reported, and causative genes have been identified for 7 of them.

In recent years, it has been reported that cases of neurodegenerative disease associated with atrophy of the cerebellar vermis and the cerebral cortex are caused by homozygous nonsense mutations of *TTC19* [GenBank:NM_017775] in Italian and Portuguese families [1,2]. *TTC19* encodes tetratricopeptide repeat domain 19 which consists of 380 amino acids. *TTC19* is involved in the assembly and activity of ubiquinol-cytochrome *c* reductase (mitochondrial respiratory chain complex III, E.C.1.10.2.2) (MRC cIII). Mammalian cIII consists of 11 subunits [3]. Among them, cytochrome *b* is the only gene encoded by mitochondrial DNA; all other subunits are encoded by nuclear DNA. Mutations in *BCS1L*[4], one of the assembly factors, are involved in most cases of cIII deficiency. According to the report mentioned above [1], the TTC19 protein is another assembly factor of cIII, and nonsense mutations in its gene cause the functional loss of cIII and, consequently, neurodegenerative disease.

Here, we performed exome sequencing on a patient clinically diagnosed with recessive SCA and identified a novel *TTC19* mutation. Her main symptoms were cerebellar ataxia and mental impairment, and magnetic resonance imaging (MRI) results were similar to those described in previous reports [1,2].

## Methods

### Patient

**Figure 1:**
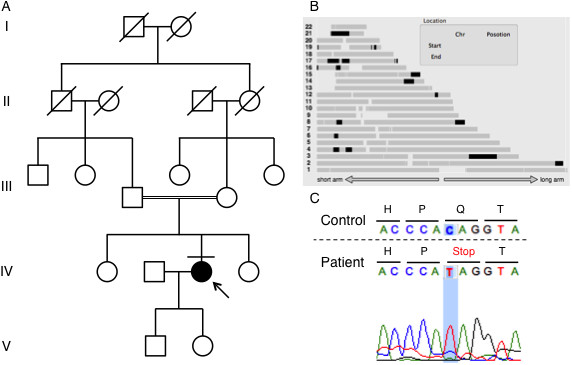
**Pedigree chart, homozygosity mapping, and capillary sequencing. A**. The patient was born to consanguineous parents who were first cousins. No other members of the family have exhibited the same symptoms. **B**. Black bar indicates IBD. Relatively long segments of IBD exist on chromosomes 3, 17, and 21. **C**. Patient has a homozygous nonsense mutation of *TTC19* (c.829C > T, p.Q277*).

**Figure 2:**
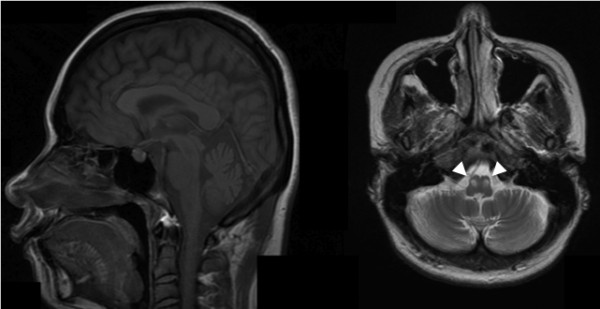
**Brain MRI. Left is T1 weighted sagittal image, and right is T2 weighted axial image.** Cerebellar atrophy was observed. Bilateral inferior olives showed T2 high intensity (arrowheads).

We performed exome sequencing on a Japanese woman born to consanguineous parents who were first cousins (Figure 1A). The 37-year-old woman noticed dysarthria at age 31. She exhibited brisk tendon reflex, ataxic gait, severe truncal ataxia, horizontal nystagmus, intellectual impairment, and pes cavus. Head MRI showed cerebellar atrophy and T1 low/T2 high intensity at the bilateral inferior olives at the age of 33 (Figure 2). Her blood lactic acid (14.0 mg/dl, normal range: 3–17 mg/dl) and pyruvic acid levels (0.62 mg/dl, normal range: 0.30-0.94 mg/dl) were normal at the age of 35 but were elevated at the age of 37 (lactic acid: 24.9 mg/dl, pyruvic acid: 1.63 mg/dl). A muscle biopsy was not performed because the patient declined consent. She began to use a wheelchair when she was 34 years old.

### Identity by descent (IBD)

SNP genotyping was performed using the Genome-Wide Human SNP Array 6.0 (Affymetrix, Santa Clara, CA, USA). Identity by descent (IBD) was calculated using Homozygosity Mapping software [5].

### Exome sequencing

Genomic DNA (gDNA) was extracted from the peripheral lymphocytes of the patients. gDNA libraries were prepared using the SeqCap EZ Human Exome Library v2.0 (Roche NimbleGen, Madison, WI, USA). Sequencing was performed with 100-bp paired-end reads on a HiSeq2000 sequencer (Illumina, San Diego, CA, USA). We used BWA (http://bio-bwa.sourceforge.net/) [6] for alignment and mapping, Samtools (http://samtools.sourceforge.net/) [7] and Picard (http://picard.sourceforge.net/) for SAM/BAM handling, GATK (http://www.broadinstitute.org/gatk/) [8] and Samtools for variant calls, and Annovar (http://www.openbioinformatics.org/annovar/) [9] for annotation. Functional predictions due to amino acid changes were estimated using PolyPhen2 (http://genetics.bwh.harvard.edu/pph2/) [10], SIFT (http://sift.bii.a-star.edu.sg/) [11], and Mutation Taster (http://www.mutationtaster.org/index.html) [12]. We evaluated copy number variants (CNVs) from exome reads using CoNIFER (http://conifer.sourceforge.net/) [13]. Control exome sequences were obtained from Japanese patients undergoing exome analysis for diseases other than SCAs. All reported genomic coordinates were in GRCh37/hg19. The identified mutations were validated with a standard polymerase chain reaction (PCR)-based amplification followed by sequence analysis with an Applied Biosystems 3130 DNA sequencer (Life Technologies, Carlsbad, CA, USA). Additionally, we enrolled 180 normal Japanese controls without any neurological disorder and screened them for this mutation with the capillary sequencer. The research procedure was approved by the ethics committee of Hiroshima University. All examinations were performed after obtaining written informed consent from the patient and control cohort for publication of this case report and any accompanying images.

## Results

### IBD

The total length of IBD is 136,462,257 bp, and the percentage of IBD is 5.2%. There are 1,283 genes in the IBD regions on the RefSeq database. *ATXN3* and *PRKCG*, causative genes of SCA3 and SCA14 respectively, are located in the IBD regions, but there are no known associated genes with recessive SCAs. Relatively long segments of IBD exist on chromosomes 3, 17, and 21 (Figure 1B). IBD analysis was used to subsequently refine the candidates.

### Exome sequencing

The results of the exome sequencing are shown in Table 1. There were 100,134,744 total reads and 90,942,725 mapped reads, and the mean coverage was 90. We obtained 98,277 variants from GATK and Samtools. We used filtering criteria consisting of zygosity, genomic position, function, open databases (dbSNP build 135, 1000 genomes and ESP 5400), regions of IBD, and population-matched controls, which resulted in several sequence variants for each sample. There were no variant candidates in the known causative genes of SCDs. From the results of filtering, 4 candidate mutations were identified in the genes of *NPM2*, *PDLIM2*, *TTC19* and *ZSCAN4* (Additional file 1: Table S1). One of these mutations was a homozygous nonsense mutation of the *TTC19* gene (GenBank:NM_017775:c.829C > T, p.Q277*), which was the most likely candidate to cause SCAs. The presence of this mutation was confirmed using capillary sequencing (Figure 1C). This *TTC19* mutation was identified in a heterozygous state in one of the 145 population-matched disease control samples but not in the 180 normal control samples. The heterozygous sample was an odontopathy patient without any neurological deficit, and the CNV data indicated no deletions or duplications of the *TTC19* gene.

**Table 1:** Result of exome sequencing

**Sequencing data**	
Total reads	100,134,744
Mapped reads	90,942,725
Mean coverage	90
**Variant**	
Total	98,277
Not found in public DBs	8,699
AA substitution	873
Homozygote	76
Not found in in-house DB	20
IBD	4
Nonsense	1

## Discussion

This report describes a *TTC19* mutation causing ataxia and metal impairment in an Asian population. According to the previous reports, all patients harbored homozygous nonsense mutations in *TTC19*, and head MRI showed characteristic findings including cerebellar atrophy and abnormal intensity at the bilateral inferior olives [1,2]. Our patient had a novel homozygous nonsense mutation of *TTC19*, and head MRI were quite similar to those previously reported. The TTC19 protein is an assembly factor of MRC cIII, which transfers electrons from coenzyme Q to cytochrome *c. TTC19* mutations lead to mitochondrial dysfunction, which causes increased levels of blood lactic acid. Simultaneously, magnetic resonance spectroscopy shows a lactic peak [1]. While the patient’s head MRI revealed abnormal features, her blood lactic acid and pyruvic acid levels were normal. However, these acid levels were elevated 6 years after the disease onset. One should consider mitochondrial abnormality and perform genetic analysis when observing such MRI characteristics. In the previous studies, no TTC19 protein was detected and the TTC19 transcript level was markedly reduced in samples with *TTC19* mutations [1,2]. However, further analysis could not be performed because we could not obtain any additional clinical specimens from the patient other than gDNA.

We identified one heterozygote for this mutation in control samples. This result indicates an allele frequency of 0.14%, but this mutation was not included in public SNP databases such as dbSNP and 1000 genomes. Therefore, the actual frequency of the mutation is even lower, which does not rule out the possibility that the mutation is a cause of recessive inheritance of the symptoms observed in the patient.

Neurodegeneration caused by mutations of *TTC19* are classified as mitochondrial complex III deficiencies (MC3DNs), including MC3DN1 [MIM:124000] [4], which is associated with compound heterozygous or homozygous mutations of the *BCS1L* gene. Clinical symptoms of MC3DN are varied, but in reports on mutations of *TTC19*, many cases exhibit neurological disorders in adulthood, and some cases present both hemiplegia and cerebellar ataxia. Pyramidal signs were not observed in our case, but intellectual dysfunction was observed. As shown in previous reports, TTC19 p.Q173Rfs*4, p.L219* [1] and p.A200Afs*8 [2] are located between the first and the second TPR domains, but p.Q277*, the novel substitution we identified is located between the second and third domains, accordingly deleting half of the TPR domains. Notably, all these mutations are nonsense mutations. Clinical symptoms were mild compared with the symptoms from previously reported cases, but determining whether mutations are associated with clinical symptoms may require a longer observation of our patient’s clinical course and the accumulation of more cases.

Regarding other variant candidates, the *NPM2* variant was predicted as benign by PolyPhen-2, Mutation Taster, and SIFT; the *ZSCAN4* variant was predicted as damaging only by SIFT. Thus, it is unlikely that *NPM2* or *ZSCAN4* is the causative mutation. The *PDLIM2* variant was predicted as disease causing by Mutation Taster and SIFT. However, it is unlikely that *PDLIM2* is the cause of SCA because PDLIM2 is expressed at low levels in the brain and is speculated to be associated with the inflammatory response of T helper 17 cells [14,15].

Several studies have implicated mutations in genes involved in mitochondrial function as a cause of SCAs [16]. For example, coenzyme Q10 deficiencies are known as diseases that lead to ataxia associated with MRC dysfunction. Thus far, there have been 6 subtypes of coenzyme Q10 deficiencies, including COQ10D4 [MIM:612016], which is also referred to as SCAR9 and is caused by homozygous or compound heterozygous mutations in *CABC1*[17]. In addition to cerebellar ataxia, coenzyme Q10 deficiencies show hypotonia, epilepsy, and muscular symptoms. Thus, mutations in the genes causing mitochondrial dysfunction show broad spectrum of clinical outcome.

## Conclusions

In conclusion, using exome sequencing, we identified a new *TTC19* mutation that is the cause of an autosomal recessive SCA. Notably, our case showed abnormal MRI findings before we detected a metabolic disorder. Genes associated with mitochondrial function cause many types of SCAs, and we should consider the genetic analysis of mitochondria-related genes when observing such characteristic clinical features including MRI abnormalities.

## Competing interests

Dr. Morino, Dr. Miyamoto, Dr. Ohnishi, Dr. Maruyama and Dr. Kawakami report no conflicts of interest.

## Authors’ contributions

HMo contributed substantially to conception and design, the acquisition of data, the analysis and interpretation of data, and the drafting of the article. RM participated in the acquisition of data, as well as the analysis and interpretation of the data. SO participated in the acquisition of samples and clinical data. HMa substantially contributed to conception and design, drafting the article, and the acquisition of funding. HK substantially contributed to conception and design, the acquisition of data, the acquisition of funding, and general supervision of the research group. All authors critically revised the article for important intellectual contents, and approved the final manuscript.

## Pre-publication history

The pre-publication history for this paper can be accessed here:

http://www.biomedcentral.com/1471-2377/14/5/prepub

## Supplementary Material

Additional file 1: Table S1 Candidate SNPs after variant filtering. Click here for file
